# Measuring Propidium Iodide Brightness as a Function of Viability: A High‐Throughput Quantitative Assessment of Hepatic Spheroids

**DOI:** 10.1111/aor.70089

**Published:** 2026-01-14

**Authors:** Michael G. Megaly, Diane Tobolt, Bat‐Erdene Namsrai, Benjamin Fisher, Anthony An‐Fa Dahm Chen, Joseph Sushil Rao, Srivasupradha Ramesh, Anudari Chimedtseren, Mark Clemens, Charles Y. Lee, John C. Bischof, Erik B. Finger

**Affiliations:** ^1^ Division of Transplantation, Department of Surgery University of Minnesota Minneapolis Minnesota USA; ^2^ Department of Mechanical Engineering University of Minnesota Minneapolis Minnesota USA; ^3^ Schulze Diabetes Institute University of Minnesota Minneapolis Minnesota USA; ^4^ Department of Biological Sciences University of North Carolina Charlotte North Carolina USA; ^5^ Department of Mechanical Engineering and Engineering Science University of North Carolina Charlotte North Carolina USA; ^6^ Institute for Engineering in Medicine University of Minnesota Minneapolis Minnesota USA

**Keywords:** bioartificial liver, fluorescence microscopy, hepatocyte spheroids, organoid, viability

## Abstract

**Background:**

Three‐dimensional (3D) hepatocyte spheroids better recapitulate liver microenvironments than monolayers, but robust, high‐throughput viability assessment remains challenging because of diffusion limits and stain penetration.

**Objective:**

To evaluate a simple imaging‐based approach that quantifies spheroid viability by measuring acridine orange (AO) and propidium iodide (PI) fluorescence and computing a PI brightness‐to‐area ratio.

**Methods:**

Rat hepatocyte spheroids were formed on a rocker, stained with AO/PI, and imaged on an inverted fluorescence microscope with fixed exposure settings. We prepared mixtures representing nominal 0%, 30%, 50%, 70%, and 100% viable spheroids and analyzed the relationship between viability and the PI brightness‐to‐area ratio. Statistical analyses included one‐way ANOVA with Tukey's HSD and simple linear regression with diagnostic checks. A urea/DNA functional assay as well as a small blinded study both served as an orthogonal validation.

**Results:**

The PI brightness‐to‐area ratio decreased with increasing percent viable cells (Pearson *r* = −0.99; *R* ^2^ = 0.98; *p* = 1.81 × 10^−4^). Residuals were approximately normal and homoscedastic. Urea/DNA strongly and positively correlated with viability (*r* = 1.00; *p* = 1.10 × 10^−4^; *R* ^2^ = 1.00) with regression equation: *y* = 9.049444 × 10^−6^ *x* + 3.036814 × 10^−5^. All blinded studies were within 10% of established viability; average difference between mean observer estimates and ground truth was +1.7 percentage points (range −3.7 to +10).

**Conclusions:**

A fixed‐setting AO/PI imaging workflow yields a rapid, accessible proxy for hepatocyte spheroid viability that correlates with a functional readout. This approach is well‐suited to high‐throughput screening and method optimization.

## Graphical abstract

The graphical abstract illustrates our rapid PI‐based spheroid viability assay: hepatocyte spheroids are formed and stained with AO/PI, imaged after a 15‐min incubation, and analyzed for PI brightness normalized to area. This metric correlates strongly with functional urea/DNA output, validating PI intensity as a fast, reliable indicator of spheroid viability.

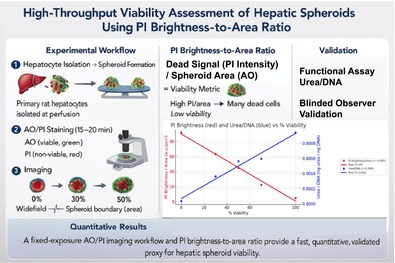

**Abbreviations**
AO: acridine orange
CPM: cycles per minute
PI: propidium Iodide
SCM: spheroid culture media
SFM: spheroid forming media

## 1 Introduction

Three‐dimensional (3D) culture systems have transformed experimental hepatology by enabling physiologically relevant liver models. Among these, hepatocyte‐derived spheroids preserve polarity, intercellular communication, and liver‐specific functions (e.g., albumin and urea secretion, cytochrome P450 activity) and exhibit superior longevity and phenotypic stability compared with 2D cultures. These attributes have driven their adoption for drug metabolism, toxicology, disease modeling, and bioartificial liver research [1, 2, 3]. They better recapitulate the in vivo liver microenvironment (compared to monolayer cultures) and exhibit enhanced survival and resistance to cryopreservation‐associated damage [4].

A central challenge is the accurate and reproducible assessment of viability within intact spheroids. Diffusion limitations create gradients of oxygen and nutrients that produce hypoxic and necrotic cores once spheroids exceed characteristic size thresholds, confounding bulk readouts and complicating certain stain penetration and quantification [5, 6]. These well‐described microenvironmental constraints underscore why methods validated in monolayers may not translate directly to 3D hepatic constructs [7, 8].

Viability is especially consequential in hepatic spheroids. Focal cell death alters diffusion, enzymatic capacity, and secretory functions that underpin the interpretation of hepatotoxicity assays and the performance of bioartificial liver systems. In the spheroid reservoir bioartificial liver (SRBAL) platform, for example, maintenance of viable hepatocyte mass is essential for detoxification and synthetic activity in vivo and in large‐animal models [9].

Multiple approaches quantify viability, each with trade‐offs. Fluorescent live/dead staining (e.g., AO/ethidium homodimer‐1) offers spatial mapping but is often qualitative rather than quantitative in aggregates of cells such as organoids, as opposed to quantitative in individual cells/monolayers. Metabolic assays (ATP, resazurin/AlamarBlue) scale well but yield bulk signals and often require protocol adaptations for 3D tissues (e.g., enhanced lysis chemistry in CellTiter‐Glo 3D or extended incubation for resazurin). High‐content imaging (confocal/light‐sheet) offers spatial resolution at the cost of throughput; and label‐free readouts (electrical impedance, Raman spectroscopy, oxygen consumption rate) enable non‐destructive monitoring but can average over heterogeneity or require specialized instrumentation [10, 11, 12, 13, 14, 15, 16]. Despite progress, no single gold‐standard viability assay has emerged for hepatic spheroids; performance depends on spheroid size, formation method (e.g., hanging‐drop, rocker‐based aggregation, microfluidics), and experimental goals. Establishing robust, standardized, and size‐aware strategies is therefore critical to ensure reproducibility and translational relevance [3, 17].

Here, we use a combination of common live/dead stains, Acridine Orange and Propidium Iodide, with fixed‐exposure fluorescence imaging and compute a PI brightness‐to‐area ratio. We hypothesize that this simple metric would inversely track percent viable cells and align with an independent functional measure (urea production), providing a practical, high‐throughput readout accessible to laboratories with standard fluorescence microscopes.

## 2 Materials and Methods

### 2.1 Hepatocyte Isolation

Sprague–Dawley rats (8–12 week‐old males) were fasted overnight, anesthetized, and heparinized. Livers were surgically recovered and flushed with 30 mL of University of Wisconsin (UW) solution, then perfused ex vivo using a normothermic perfusion system with 1× Krebs‐Henseliet Buffer for 10 min, followed by 10 min of collagenase perfusion. Hepatocytes were released by gentle disruption of the liver capsule, filtered, and washed. Trypan Blue exclusion showed 96%–98% viability. All procedures were approved by the University of Minnesota IACUC (protocol #2504‐42888A).

### 2.2 Spheroid Formation

Hepatocytes were plated at a density of 1 × 10^6^ cells/mL in 20 mL of spheroid formation medium (SFM) and plated into rectangular Mayo dishes (10 × 8 × 2 cm). The SFM consisted of 887 mL of Nanopure water, Williams' E medium powder (1×), 2.2 g sodium bicarbonate, 2 mL penicillin/streptomycin, 10 mL fetal bovine serum, and 0.5 mL heparin. The dishes were placed on an IBI Scientific Hi‐Lo Rocker (model ROCAA115S; IBI Scientific, Dubuque, IA, USA) set at a 7°–11° tilt and nine cycles per minute. Cultures were maintained in a Thermo Scientific Napco Series 8000 DH water‐jacketed CO_2_ incubator (Thermo Fisher Scientific, Waltham, MA, USA) at 37 C with 5% CO_2_ and 95% humidity. After 24 h spheroids are formed and visible on microscopy. At that 24 h mark, the SFM is replaced with spheroid culture medium (SCM), composed of Williams' E medium (1 L prepared from powder), 2.2 g sodium bicarbonate, 3.6 g HEPES, 1 mL heparin, and 28 mL of a spheroid supplement solution containing bovine serum albumin, linoleic acid, human epidermal growth factor (EGF), dexamethasone, amino acids, and additional micronutrients.

### 2.3 Imaging and Analysis

**FIGURE 1:**
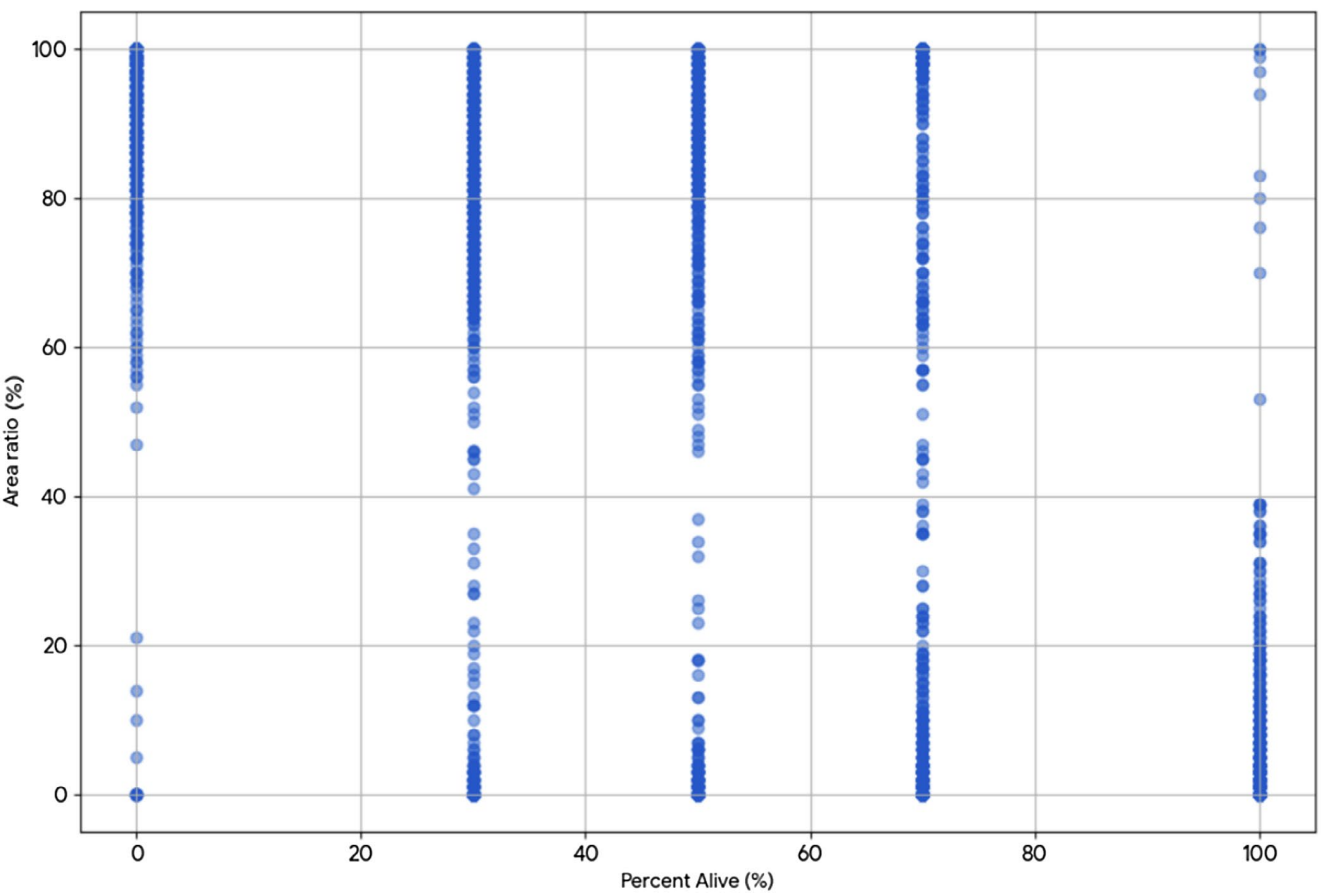
Scatter plot of all data points. Each point represents a single spheroid's PI brightness‐to‐area ratio (*y*‐axis) versus nominal percent alive (*x*‐axis), representing thousands of data points. [Color figure can be viewed at wileyonlinelibrary.com]

**FIGURE 2:**
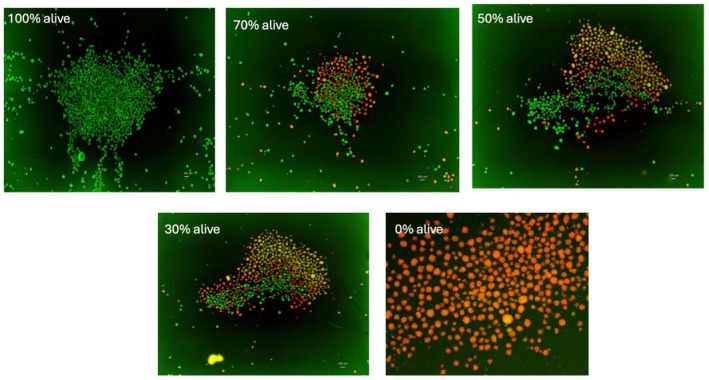
AO/PI under fluorescence microscopy based on percent of alive sample. Spheroids stained at 4× magnification with AO (5 μg/mL) and PI (10 μg/mL) for 20 min at room temperature and imaged on an inverted fluorescence microscope with fixed exposure settings (green/AO 0.2 s; red/PI 0.167 s). Panels illustrate increased PI‐positive (non‐viable) signal and decreased AO‐positive (viable) signal as overall viability declines. Images were acquired and displayed using identical settings; AO, acridine orange; PI, propidium iodide. From top to bottom and left to right, the samples are of 100% viability, 70%, 50%, 30%, 0%. [Color figure can be viewed at wileyonlinelibrary.com]

Spheroids were imaged on Day 3 after hepatocyte isolation using a Keyence BZ‐X810 inverted fluorescence microscope (Keyence Corporation of America, Itasca, IL, USA) (Figures 1 and 2). Acridine Orange (5 μg/mL) and Propidium Iodide (10 μg/mL) were applied to evaluate spheroid viability. A 100 μL sample of spheroids was incubated with 80 μL of the AO/PI solution in an 8‐well Ibidi slide (μ‐Slide 8 Well, ibiTreat, Cat. 80 826; Ibidi GmbH, Gräfelfing, Germany) for 15–20 min at room temperature in the dark. Exposure settings were kept constant for all images (green channel: 0.2 s; red channel: 0.167 s), as these conditions provided optimal clarity for analysis. Using the Keyence analysis software, AO and PI signals were quantified. Total spheroid area was measured from AO fluorescence, while PI intensity was used to identify non‐viable regions in the subtraction analysis (Figure 2).

### 2.4 Preparation of Viability Mixtures

To construct reference mixtures, we combined spheroids deemed 100% alive (control spheroids) and spheroids that were rendered nonviable by liquid nitrogen freeze and slow thaw to approximate 0% viability. Mixtures targeting 30%, 50%, and 70% viability were prepared volumetrically (e.g., 350 μL “alive” + 150 μL “dead” for 70% viable). Each condition was prepared in triplicate across multiple independent isolations.

### 2.5 Urea Assay

For measurement of urea production, we used the QuantiChrom Urea Assay Kit (QuantiChrom Urea Assay Kit, DIUR‐500; BioAssay Systems, Hayward, CA). One milliliter of spheroid suspension of varying viabilities was washed with phosphate‐buffered saline (PBS) and then incubated for 2 h in arginase‐free Krebs–Henseleit–Hepes (KHB) buffer as described by Peters et al. [18] Following incubation, spheroids were transferred to 1.6‐mL Eppendorf tubes, the supernatant was collected, and the spheroid pellet was lysed via ultrasonication for downstream DNA quantification to normalize urea production. DNA content was measured using the Quant‐iT PicoGreen dsDNA Assay Kit (Invitrogen, Thermo Fisher Scientific; Cat. P11496) according to the manufacturer's instructions.

### 2.6 Statistical Analysis

Analyses were performed using Python (version 3.x). For each percent viable group (0%, 30%, 50%, 70%, 100%), descriptive statistics were calculated for the PI brightness‐to‐area ratio. A one‐way ANOVA tested for differences among groups (*α* = 0.05), followed by Tukey's HSD for pairwise comparisons. Simple linear regression quantified the relationship between percent viable (predictor) and PI brightness‐to‐area ratio (outcome). Model diagnostics included visual assessment of residual normality (histograms and Q–Q plots) and homoscedasticity (residuals versus fitted values). A urea/DNA assay served as functional validation, with linear regression and correlation reported similarly.

### 2.7 Blinded Observer Validation

**FIGURE 3:**
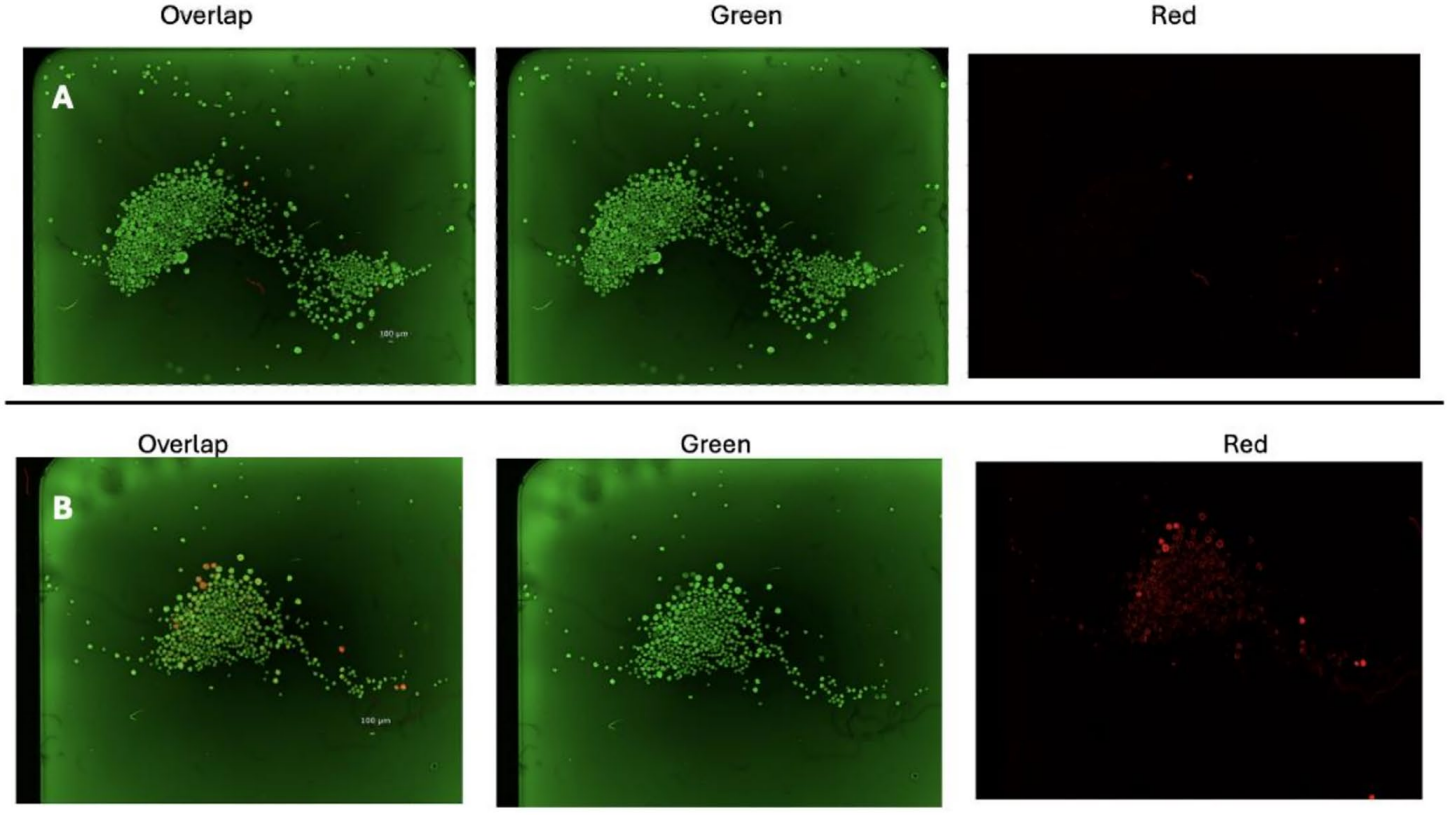
Representative blinded images used for observer estimation of spheroid viability. Two example hepatocyte spheroid fields stained with Acridine Orange (AO; green, viable cells) and Propidium Iodide (PI; red, non‐viable cells) are shown under 4× magnification on a Keyence BZ‐X810 fluorescence microscope first column demonstrates both AO and PI, second column demonstrates only AO‐green channel, third column demonstrates only PI‐red channel. Observers, blinded to experimental conditions and reference values, were asked to estimate the percent viability in each image. Upper panel (A): True viability = 99%; observer estimates were 99%, 90%, and 97%. Lower panel (B): True viability = 85%; observer estimates were 95%, 90%, and 95%. These examples illustrate the visual basis for observer assessment and highlight both the overall agreement and the rater‐specific variation seen in the blinded validation study. [Color figure can be viewed at wileyonlinelibrary.com]

To provide an additional validation of image‐based viability, we conducted a blinded visual estimation study. Five representative AO/PI‐stained spheroid images spanning a range of viabilities were selected. These spheroids were derived from control groups that had been subjected to varying durations of vigorous agitation to generate heterogeneous injury patterns within individual spheroids. Three independent observers, blinded to experimental conditions and reference values, were asked to estimate the percent alive in each image. Their estimates were compared against the known brightness from the microscope, which served as the ground truth for percent alive (Figure 3).

### 2.8 Assumption Validation

Prior to performing the parametric analyses, the assumptions of normality and homoscedasticity were rigorously checked. Residuals appeared approximately normal by Q‐Q plots and histograms. A residuals‐versus‐fitter plot showed no funneling or trend, supporting homoscedasticity and the appropriateness of ANOVA and linear regression for these data (Figure 2).

## 3 Results

### 3.1 PI Brightness‐To‐Area Ratio Tracks Viability

**FIGURE 4:**
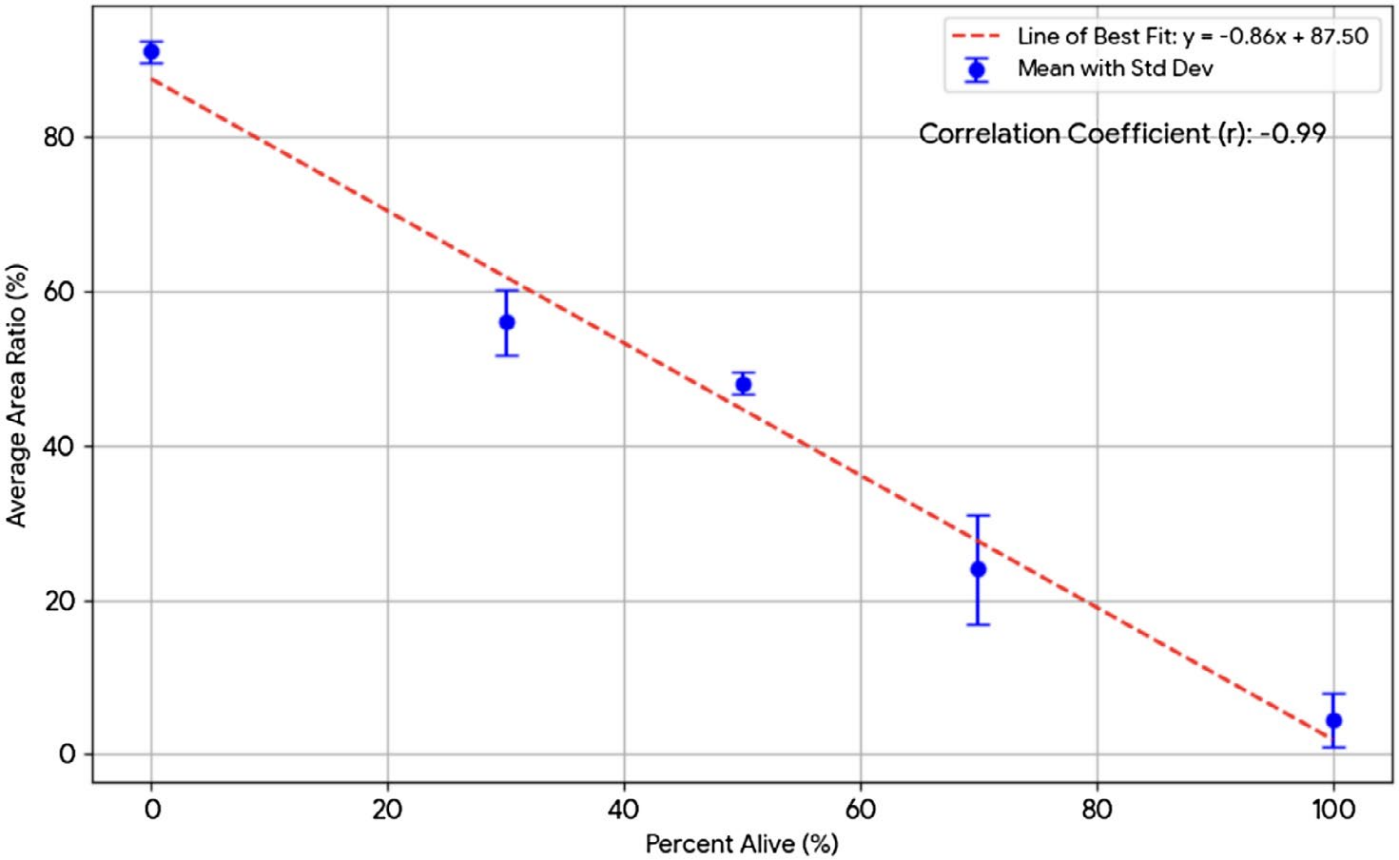
PI brightness area ratio/percent alive sample, line of best fit. Group means of the PI brightness‐to‐area ratio plotted against nominal percent alive (0%, 30%, 50%, 70%, 100%). Exposure was fixed for all images to enable direct comparison across groups. A monotonic decrease in PI/area is observed with increasing viability, consistent with reduced non‐viable cell burden at higher percent alive. Error bars denote SD. [Color figure can be viewed at wileyonlinelibrary.com]

The mean PI brightness‐to‐area ratio decreased monotonically as the nominal percent viable samples increased, with group means summarized in Table 1 and a representative image in Figure 4. A one‐way ANOVA demonstrated a significant effect of percent viability on the PI brightness‐to‐area ratio (*p* < 0.001). Tukey's HSD identified multiple significant between‐group differences. A simple linear regression demonstrated a very strong inverse association between the two variables (Pearson *r* = −0.99, *R* ^2^ = 0.98, *p* = 1.81 × 10^−4^). This highlights a robust and predictable inverse relationship between the two measured parameters. A scatterplot of all observations is shown in Figure 1.

**TABLE 1:** Group means (±SD) for PI brightness‐to‐area ratio across nominal viability levels (0%, 30%, 50%, 70%, 100% alive).

% Alive	PI brightness/area ratio 1	PI brightness/area ratio 2	PI brightness/area ratio 3
100%	5 (±12)	2 (±7)	7 (±11)
70%	19 (±36)	29 (±44)	19 (±35)
50%	38 (±43)	47 (±46)	49 (±45)
30%	78 (±34)	53 (±41)	59 (±44)
0%	93 (±11)	90 (±17)	92 (±17)

*Note:* Values are reported as mean ± SD of the whole‐spheroid propidium iodide (PI) brightness normalized to spheroid area, measured under fixed exposure (green channel 0.20 s, red channel 0.167 s) after acridine orange (AO; 5 μg/mL)/PI (10 μg/mL) staining.

### 3.2 Functional Assay Validation—Urea Production

**FIGURE 5:**
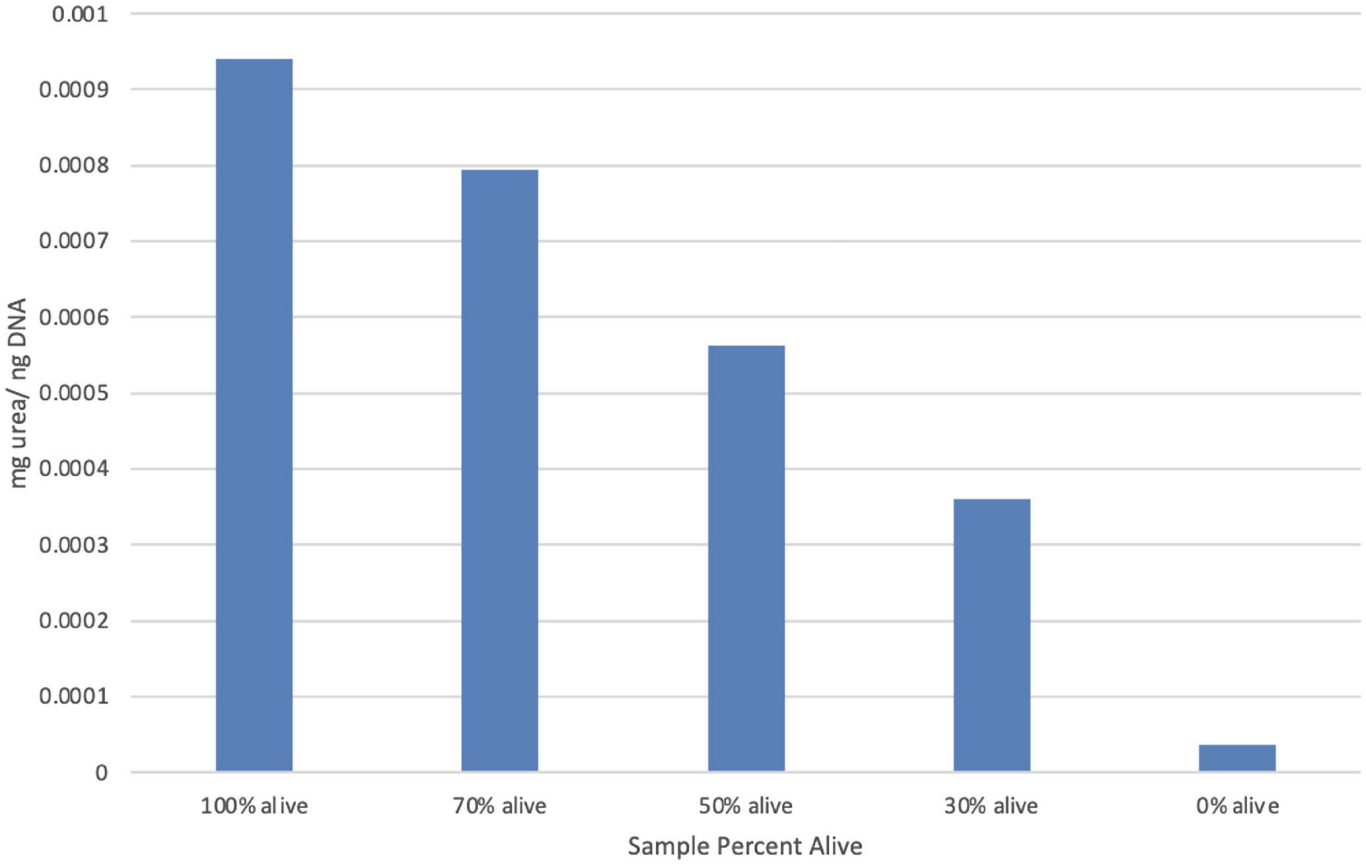
Urea production/DNA based on percent of living sample. Urea production normalized to DNA content (*y*‐axis) decreased with decreasing viability (percent alive) (*x*‐axis). DNA, deoxyribonucleic acid. [Color figure can be viewed at wileyonlinelibrary.com]

To further validate the physiological relevance of the PI area ratio findings, a functional assay measuring urea production, normalized to DNA quantity, was conducted. Urea production normalized to DNA content increased with viability and demonstrated a nearly perfect linear relationship with percent viable (*r* = 1.00, *p* < 0.001, *R* ^2^ = 1.00). The regression equation was *y* = 9.05 × 10^−6^ *x* + 3.04 × 10^−5^. These results corroborate the biological relevance of the image‐based viability metric (Figure 5), providing a validation for the viability seen in the PI brightness‐to‐area ratio model demonstrated here.

### 3.3 Blinded Observer Validation

Observer estimates tracked closely with the true percent viability (Figure 3). Across five images, the average difference between mean observer estimates and ground truth was +1.7 percentage points (range −3.7 to +10). Rater‐specific biases were observed: one rater consistently overestimated (+5.8%), one underestimated (−8.2%), and one was closely calibrated (+1.0%). Despite these tendencies, all estimates were within ~10 percentage points of the true values. Although formal significance testing was limited by small sample size, the blinded analysis demonstrates that independent observers can approximate viability from AO/PI images with reasonable fidelity, reinforcing the interpretability of the imaging‐based approach.

## 4 Discussion

This study evaluated a simple fluorescence‐imaging metric—the PI brightness‐to‐area ratio—as a high‐throughput estimate for hepatocyte spheroid viability. The metric was inversely correlated with viability and agreed closely with an independent functional readout (urea production). Together, these findings support the use of fixed‐exposure AO/PI imaging as a rapid, accessible approach for large‐scale experiments such as cryopreservation optimization or drug screening.

Measuring spheroid viability is a challenge and has been addressed using a myriad of methods, none of which are perfect and universally applicable. Other methods include alamar blue, which may be used as a standard colorimetric method based on resazurin reduction in 2D cell cultures [19]. However, it was found not to be applicable in 3D spheroid/microtissues due to tight cell–cell junctions which affect uptake and diffusion kinetics. Numerous assays have been used, including ATP assays; however, these may not accurately reflect viability of cells that have not yet recovered ATP levels post treatment. Assays are commonly normalized using a secondary metric (e.g., DNA quantity) which also introduces further possible error. Yet another method of assessing viability is dissociation of the spheroid followed by staining or other assessments [20] of the resulting cell suspension. However, there is some difficulty with dissociation, and the process may be harmful to the final viability if cells are damaged due to excess time in dissociation media or rough agitation. It is also possible for the spheroids to insufficiently dissociate.

### 4.1 Interpretation of Area Ratio Findings

Propidium Iodide (PI) is membrane‐impermeant and labels the nuclei of non‐viable cells; therefore, a larger PI‐positive area indicates greater cell death within a spheroid. The inverse relationship observed between percent viability and the PI brightness‐to‐area ratio is consistent with this mechanism. Although we do not directly measure viability on a per‐cell basis, the data can be interpreted as representative of that metric. In contrast, other approaches often report viability per spheroid, where a spheroid is considered viable as long as the majority of its cells remain alive.

### 4.2 Validation With Functional Assay (Urea Production)

Hepatocytes are the primary site of urea synthesis, making urea production a direct and sensitive indicator of hepatic metabolic function and viability. The results from this assay showed a remarkably strong positive linear correlation between percent alive and the urea/DNA ratio (*r* = 1.00, *p* < 0.001). This near‐perfect correlation unequivocally demonstrates that as spheroid viability increases, their functional capacity to produce urea also increases proportionally. Crucially, this robust functional data provides independent confirmation for the reliability of the PI brightness area ratio. The alignment between the morphological (PI exclusion) and functional (urea synthesis) assessments strongly supports that the PI area ratio is a valid surrogate marker for hepatic spheroid viability.

### 4.3 Validation With Blinded Study

Blinded observer estimation provided an additional layer of validation. Independent raters, without knowledge of the ground truth, were able to approximate spheroid viability from AO/PI images within ~10 percentage points. While small in scale, this experiment highlights that viability assessments derived from fluorescence patterns are interpretable and biologically plausible even by human inspection. Importantly, rater‐specific biases underscore the value of quantitative, standardized image analysis over subjective judgment. Together with the functional urea assay, these findings strengthen confidence that the PI brightness‐to‐area ratio reflects true biological viability.

### 4.4 Advantage of the PI Method

In our study, propidium iodide (PI) brightness provided several advantages over traditional viability metrics used in 3D hepatocyte cultures. Conventional assays—such as Trypan Blue exclusion, LDH release, ATP quantification, or resazurin reduction—are optimized for 2D monolayers or dissociated cells and often fail to capture spatial heterogeneity of cell death within intact spheroids. Metabolic and enzymatic assays also provide bulk readouts that obscure localized injury, and they require multiple reagents, extended incubation periods, or spheroid disruption. In contrast, PI brightness directly quantifies nuclear fluorescence in membrane‐compromised cells, allowing sensitive and reproducible detection of apoptosis and necrosis while preserving the 3D architecture. The method is also highly time‐efficient: spheroids require only a 15–20 min incubation period with AO/PI, and imaging takes seconds per sample on a standard fluorescent microscope, enabling rapid, near‐real‐time viability assessment. Together, these advantages make PI brightness a practical, physiologically relevant, and high‐throughput approach for evaluating spheroid viability lending itself to automation with consistent exposure settings and batch analysis.

### 4.5 Limitations

While highly promising, this study has certain limitations. Firstly, the “0%” and “100%” viability references are approximations—some cells in the “dead” group may remain viable and vice versa—introducing bounded error acceptable for a screening metric. The assumption that fresh control spheroids are all “alive” and frozen and thawed ones all “dead” may not be absolutely true. But, we use these controls for reference values, and relative viability to those controls will still be valid. Secondly, PI penetration and optical sectioning may limit detection in very large spheroids; while our diameters (~100–150 μm) are within typical imaging depths, larger constructs may require clearing, sectioning, or light‐sheet/two‐photon imaging. Finally, stain concentration and incubation time can influence signal; modest optimization may be needed across cell types and platforms. However, when it comes to cryopreservation or drug testing, the typical area of highest injury is the cortical layers of the spheroid, which are exposed to the highest concentration of the solution/agent of interest. Therefore, hepatocytes closer to the center (within a < 200 μm diameter spheroid) are likely to be in less contact; meaning if the cortical layer has minimal PI brightness, the deeper hepatocytes are also likely to be alive and well.

### 4.6 Future Directions

Integrating automated segmentation, radial intensity profiling, and plate‐level analytics could further enhance precision and throughput. Extending validation across additional hepatocyte sources (e.g., PHH, HepaRG), co‐cultures, and chronic exposure paradigms would strengthen generalizability.

## 5 Conclusion

A fixed‐exposure AO/PI fluorescence imaging workflow provides a rapid, high‐throughput, and biologically meaningful proxy for hepatocyte spheroid viability. The PI brightness‐to‐area ratio demonstrates strong, directionally consistent relationships with percent viable cells, aligns with urea/DNA functional output, and is broadly interpretable even by blinded human observers. These convergent lines of validation support its utility for screening and method optimization in 3D hepatic models such as those testing novel drugs, toxicities, attempting cryopreservation, or investigation into the utility of hepatic spheroids in BALs.

## Author Contributions

**Michael G. Megaly:** conceptualization. **Michael G. Megaly** and **Diane Tobolt:** methodology. **Michael G. Megaly** and **Erik B. Finger:** formal analysis. **Michael G. Megaly**, **Diane Tobolt**, and **Mark Clemens:** writing. **Michael G. Megaly**, **Diane Tobolt**, **Bat‐Erdene Namsrai**, **Benjamin Fisher**, **Joseph Sushil Rao**, **Srivasupradha Ramesh**, and **Anudari Chimedtseren:** review and editing. **Mark Clemens**, **Charles Y. Lee**, **John C. Bischof**, and **Erik B. Finger:** supervision. All authors have read and agreed to the published version of the manuscript.

## Funding

This work was supported by NIH grants DK117425 (Erik B. Finger, John C. Bischof), DK132211 (Erik B. Finger, John C. Bischof), and DK131209 (Erik B. Finger, John C. Bischof), NSF grant EEC‐1941543 (Erik B. Finger, John C. Bischof), and a gift from the Biostasis Research Institute funded in part through contributions from LifeGift, Nevada Donor Network, Lifesource, Donor Network West, and Lifebanc.

## Ethics Statement

The study was approved by the University of Minnesota IACUC (protocol #2504‐42888A).

## Conflicts of Interest

The authors declare no conflicts of interest.

## Data Availability

All Data can be made available on request.
